# QiShenYiQi Pills, a Compound Chinese Medicine, Prevented Cisplatin Induced Acute Kidney Injury via Regulating Mitochondrial Function

**DOI:** 10.3389/fphys.2017.01090

**Published:** 2017-12-21

**Authors:** Li Zhou, Xiao-Hong Wei, Chun-Shui Pan, Li Yan, You-Yu Gu, Kai Sun, Yu-Ying Liu, Chuan-She Wang, Jing-Yu Fan, Jing-Yan Han

**Affiliations:** ^1^Department of Integration of Chinese and Western Medicine, School of Basic Medical Sciences, Peking University, Beijing, China; ^2^Tasly Microcirculation Research Center, Peking University Health Science Center, Beijing, China; ^3^Key Laboratory of Microcirculation, State Administration of Traditional Chinese Medicine of the People's Republic of China, Beijing, China; ^4^Key Laboratory of Stasis and Phlegm, State Administration of Traditional Chinese Medicine of the People's Republic of China, Beijing, China; ^5^State Key Laboratory of Core Technology in Innovative Chinese Medicine, Beijing, China

**Keywords:** acute renal failure, apoptosis, energy metabolism, astagalus membranaceus, *Salvia miltiorrhiza*

## Abstract

Nephrotoxicity is a serious adverse effect of cisplatin chemotherapy that limits its clinical application, to deal with which no effective management is available so far. The present study was to investigate the potential protective effect of QiShenYiQi Pills (QSYQ), a compound Chinese medicine, against cisplatin induced nephrotoxicity in mice. Pretreatment with QSYQ significantly attenuated the cisplatin induced increase in plasma urea and creatinine, along with the histological damage, such as tubular necrosis, protein cast, and desquamation of epithelial cells, improved the renal microcirculation disturbance as indicated by renal blood flow, microvascular flow velocity, and the number of adherent leukocytes. Additionally, QSYQ prevented mitochondrial dysfunction by preventing the cisplatin induced downregulation of mitochondrial complex activity and the expression of NDUFA10, ATP5D, and Sirt3. Meanwhile, the cisplatin-increased renal thiobarbituric acid-reactive substances, caspase9, cleaved-caspase9, and cleaved-caspase3 were all diminished by QSYQ pretreatment. In summary, the pretreatment with QSYQ remarkably ameliorated the cisplatin induced nephrotoxicity in mice, possibly via the regulation of mitochondrial function, oxidative stress, and apoptosis.

## Introduction

Cis-diamminedichloroplatinum (cisplatin; DDP) is a highly effective chemotherapeutic drug against a wide range of cancers including testicular, ovarian, bladder, head and neck, uterine cervical carcinoma, non-small cell lung carcinoma, etc. (Dilruba and Kalayda, 2016). Unfortunately, its therapeutic effectiveness is limited by severe side effects in normal tissues. Nephrotoxicity is a frequent adverse effect which occurs in up to one-third of patients undergoing DDP therapy in spite of the intensive prophylactic measures used, such as aggressive hydration and forced diuresis (Arany and Safirstein, 2003; Sánchez-González et al., 2011). The antineoplastic effect of DDP has been principally ascribed to its ability to form intra- and interstrand DNA crosslinks that interfere with DNA replication and synthesis, and lead to cell death (Marullo et al., 2013). Despite intensive investigation, the mechanisms underlying DDP-induced nephrotoxicity are not fully understood. Therefore, the discovery of new, effective treatments against DDP-induced nephrotoxicity is in need in order to increase the clinical utility of this drug.

DDP is known to accumulate in mitochondria and inhibit the activity of all complexes involved in oxidative phosphorylation, resulting in excessive reactive oxygen species (ROS) formation, and impairment in ATP synthesis (Nowak, 2002; Marullo et al., 2013). It causes an activation of the intrinsic mitochondrial pathway of apoptosis by the release of caspase9 mediators (Park et al., 2002; Jiang et al., 2009). Thus, preservation of mitochondrial function and inhibition of renal tissue apoptosis via mitochondrial pathway may therefore protect against cisplatin nephrotoxicity (Yang et al., 2014). QiShenYiQi Pills (QSYQ) is a compound Chinese medicine composing of Radix Astragali, *Salvia miltiorrhiza, Panax notoginseng*, and Rosewood, which was approved by the State Food and Drug Administration of China in 2003 for treatment of cardiac dysfunction (Wang et al., 2011). Our previous studies demonstrated that QSYQ ameliorates pressure overload induced cardiac hypertrophy (Chen Y. Y. et al., 2015), doxorubicin induced cardiac dysfunction (Tang et al., 2013), cardiac ischemia reperfusion injury and cardiac fibrosis in rat cardiac hypertrophy (Li et al., 2012; Lin et al., 2013; Chen J. R. et al., 2015). The mechanism behind the observed effect of QSYQ may be closely related to the recovery of energy supply, in addition to the reduction of oxidative stress (Li et al., 2012; Lin et al., 2013; Tang et al., 2013; Chen J. R. et al., 2015; Chen Y. Y. et al., 2015; Han et al., 2017) The present study was conducted to explore the effect of QSYQ on cisplatin induced kidney injury, and the possible implication of the mitochondrial function modulation.

## Materials and methods

### Animal model and drug administration

Male C57BL/6 mice (8–10 week old) were purchased from the Animal Center of Peking University with the certificate number SCXK 2006-0008. The mice were housed in cages at temperature 22 ± 2°C, humidity 40 ± 5%, under a 12-h light/dark cycle, and received standard diet and water *ad libitum*. The experimental procedures were in accordance with the recommendations of U. K. Animals (Scientific Procedures) Act, 1986 and associated guidelines, EU Directive 2010/63/EU for animal experiments. All animals were handled according to the guidelines of the Peking University Animal Research Committee. The experimental protocol was approved by the Committee on the Ethics of Animal Experiments of Peking University Health Science Center (LA2016307). QSYQ (Batch number: 140612) was obtained from Tasly Pharmaceutical Co. Ltd. (Tianjin, China), which was produced according to the guidelines of Good Manufacturing Practice and Good Laboratory Practice, and the content of its major components was determined by HPLC finger print (Lin et al., 2013). It was dissolved in distilled water to make a solution at concentration of 200 mg/ml before experiment. DDP was purchased from Sigma-Aldrich Co. LLC., which was freshly prepared in saline at 1 mg/ml before use and injected intraperitoneally into mice at a dose of 20 mg/kg to induce acute kidney injury (Jia et al., 2011; Wang et al., 2015). The animals were divided into four groups: control (CTR+vehicle; *N* = 18), QSYQ pretreatment alone (CTR + QSYQ; *N* = 18), DDP induced kidney injury (DDP + vehicle; *N* = 18), and DDP induced kidney injury with QSYQ pretreatment (DDP + QSYQ; *N* = 18) groups. The mice were pretreated for 48 h with distilled water (CTR + vehicle and DDP + vehicle) or QSYQ (CTR + QSYQ and DDP + QSYQ) at a dose of 3.6 g/kg/d through gavage. Then, the mice received an i.p. injection of DDP (DDP + vehicle and DDP+QSYQ) or saline (CTR+QSYQ and DDP+QSYQ), and followed by administration of distilled water (CTR+vehicle and DDP+vehicle) or QSYQ (CTR+QSYQ and DDP+QSYQ) once a day by gavage for subsequent 3 days.

### Plasma urea and creatinine

Plasma urea (BUN) and creatinine levels were determined to assess the renal function. After mice euthanasia 72 h after DDP treatment, blood samples were obtained from the inferior vena cave and anticoagulated with heparin (20 unit/ml blood). The plasma was isolated by centrifugation. The plasma BUN and creatine were analyzed using urea assay kit (Biosino Bio-Technology and Science Inc., Beijing, China) and creatine assay kit (Leagene Biotechnology Co.Ltd., Beijing, China) according to the instruction of the manufacture.

### Periodic acid-Schiff staining

Mice kidneys were fixed in 4% paraformaldehyde (PFA), embedded in paraffin, sectioned at 4 μm, and stained with periodic acid-Schiff (PAS) by standard method. The degree of tissue damage was scored based on the percentage of damaged tubules as previously described: 0, no damage; 1, <25%; 2, 25–50%; 3, 50–75%; 4, >75% (Jia et al., 2011).

### Renal blood flow

Renal blood flow (RBF) was measured by using Laser-Doppler Perfusion Imager (PeriScan PIM3 System; PERIMED, Stockholm, Sweden) equipped with a computer at 72 h after the DDP treatment. For this purpose, a 10- to 15-mm lateral incision was made dorsally and the kidney was exteriorized after assuring adequate anesthesia. A computer controlled optical scanner directed a low-powered He–Ne laser beam over the exposed kidney. The scanner head was positioned in parallel to the surface of kidney at a distance of 18 cm. At each measuring site, the beam illuminated the tissue to a depth of 0.5 mm (Lin et al., 2013). A color coded image to denote specific relative perfusion level was displayed on a video monitor, and all images were evaluated with the software LDPIwin 3.1 (PeriScan PIM3 System; PERIMED, Stockholm, Sweden).

### The average renal microvascular flow velocity and the number of adherent leukocytes

The kidney was exposed as stated for assessment of RBF. A 30-gauge cannula was inserted into the femoral vein for dye infusion (Dunn et al., 2002). The blood vessels were labeled with 0.1 ml of Alexa fluor 647-bovine serum albumin (BSA) (1 mg/ml) (Biosynthesis Biotechnology Co.Ltd., Beijing, China) by intravenous injection. Images acquired with longitudinal line scans were binarized, allowing detection of individual red blood cells (RBCs) and measurements of RBC flow velocity. The microvascular blood flow was measureed as described by Chaigneau et al. (2003) and Tang et al. (2015). In brief, in each binarized image, RBCs were unlabeled and drawn oblique shadows near the beginning and the end of the capillary length scanned by the laser. The X axis represents the distance traveled, the Y axis represents the the arrival time and the slope represents RBC flow velocity. Ten to 15 microvessels of 6–8 μm diameter were measured per animal. To evaluate the leukocyte adhesion, 0.05 ml of the acridine orange (1 mg/ml) was administrated via the left femoral vein for selective staining of leukocytes *in vivo* (Cahoon et al., 2014). The renal microvessels were observed under irradiation at a wavelength of 488 and 647 nm by Leica TCS-SP8 confocal microscope. The number of leukocyte adhesion in renal cortex was counted in five consecutive vision.

### Kidney tissue ATP content, thiobarbituric acid-reactive substances levels, and mitochondrial complexes activity

ComplexI, II, and IV activity was analyzed by ComplexI, II, and IV enzyme activity microplate assay kit (Abcam, Cambridge, UK). The measurement of thiobarbituric acid-reactive substances (TBARS) in the mouse kidney tissue was based on the formation of malondialdehyde by using a commercially available TBARS Assay kit (Cayman Chemical, Michigan, USA) according to the manufacturer's instructions. ATP content was detected by using mice ATP ELISA Kit (Nanjing Jiancheng Bioengineering Institute, Jiangsu, China). All plates were analyzed on MULTISKAN MK3 enzyme micro-plate reader (Thermo Fisher Scientific Inc., Illinois, USA), according to manufacturer's instruction.

### Double staining of F-actin and terminal deoxynucleotidyl transferase-mediated dUTP nick end labeling

Kidney was fixed in 4% PFA solution for 48 h for preparation of paraffin section (4 μm). Sections were incubated overnight at 4°C with phalloidine (Invitrogen, California, USA) for F-actin staining and then subjected to TUNEL staining using a cell death detection kit (Roche, Basel, Switzerland), according to the manufacture's instruction, and the nuclei were labeled with hoechst 33342. Five fields were selected from renal cortex for each section at × 40 magnification of objective, and observed with a Laser Scanning Confocal Microscope (TCS SP5, Leica, Mannheim, Germany). The numbers of the TUNEL-positive cells in the five fields were counted, and the average was calculated.

### Western blotting assay

The renal cortex was lysed and protein concentration was determined by bicinchoninic acid (BCA) protein assay kit (Applygen Technologies, Beijing, China). Proteins from renal cortex lysates were denatured in boiling water for 10 min, separated by sodium dodecyl sulfate (SDS)-polyacrylamide gel electrophoresis, and transferred onto nitrocellulose membranes. The blots were blocked overnight with 5% skim milk in Tris-buffered saline. The membrane was incubated overnight at 4°C with following antibodies: the antibody against NDUFA10 and ATP5D (Santa Cruz Biotechnology, Santa Cruz, California, USA), the antibody against ATP synthase α and ATP synthase β (Becton, Dickinson and Company, BD, New Jersey, USA), the antibody against SDHA, Sirt3, caspase9, cleaved-caspase9, caspase3, cleaved-caspase3, Bax, Bcl2, and β-actin (Cell Signaling Technology, CST, Massachusetts, USA). After being washed with TBST, blots were incubated with respective peroxidase-conjugated secondary antibodies and visualized with ECL kits.

### Statistical analysis

All parameters were expressed as mean±S.E. Statistical analysis was performed using one-way ANOVA followed by Tukey test for multiple comparisons. A probability of less than 0.05 was considered to be statistically significant.

## Results

### QSYQ attenuates DDP-induced renal dysfunction

**Figure 1:**
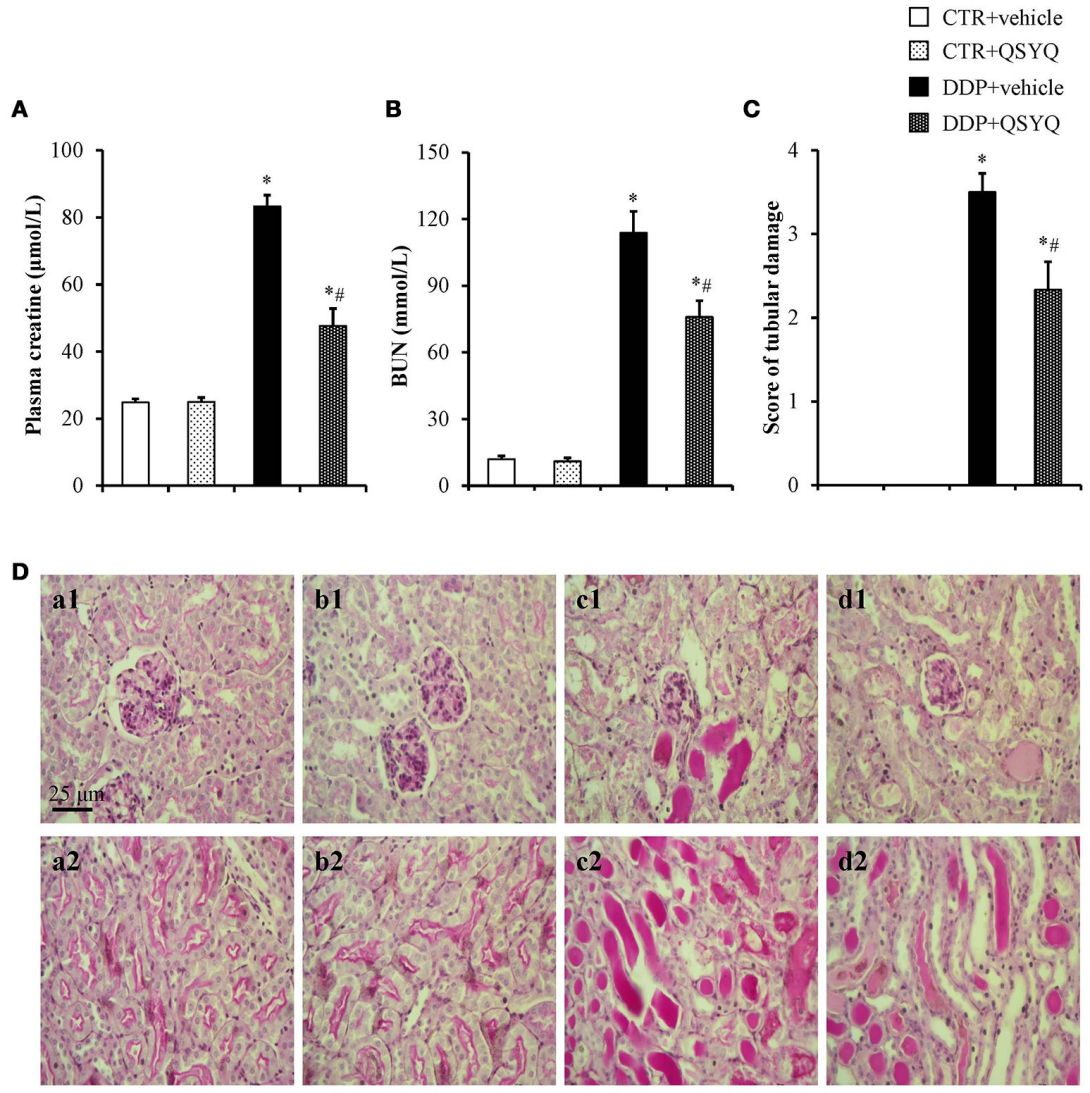
QSYQ protected the DDP-induced kidney injury. **(A)** Plasma level of creatine in different groups. **(B)** Plasma level of BUN in different groups. **(C)** Renal injury score in different groups. CTR+vehicle: control plus vehicle group (*N* = 6); CTR+QSYQ: control plus QSYQ group (*N* = 6); DDP+vehicle: DDP plus vehicle group (*N* = 6); and DDP+QSYQ: DDP plus QSYQ group (*N* = 6). ^*^P < 0.05 vs. CTR+vehicle group; ^#^*P* < 0.05 vs. DDP+vehicle group. Data are mean ± SE. **(D)** Representative micrographs (magnification ×200) of periodic acid-Schiff staining exhibiting DDP-induced severe tubular necrosis(**a1–d1**; cortex) and a large number of protein cast (**a2–d2**; the boundary between the cortex and the medulla) in CTR+vehicle **(a)**, CTR+QSYQ **(b)**, DDP+vehicle **(c)**, DDP+QSYQ **(d)** group, respectively. Bar = 25 μm.

The renal function was assessed by the plasma BUN and creatinine levels(Figures 1A,B). There was no difference between CTR+vehicle group and CTR+QSYQ group. The DDP injection induced severe renal dysfunction as indicated by a significantly increase of plasma creatinine and BUN. Remarkably, both the DDP-increased plasma creatine and BUN levels were significantly protected in the DDP+QSYQ group.

### QSYQ attenuates DDP-induced renal tubular injury

To assess renal tissue damage histologically, PAS-stained sections of kidney specimens were prepared from mice 72 h post DDP administration (Figure 1D). The structure of mouse renal tissues in the CTR+vehicle group and CTR+QSYQ group was normal (Figures 1D,a1,a2,b1,b2). Following the DDP treatment, the mice in the DDP+vehicle group displayed apparent renal pathological changes, characterized by the severe tubular necrosis and a large number of protein cast (Figures 1D,c1,c2). The pathological damages of renal tissues in the DDP+QSYQ group were obviously improved (Figures 1D,d1,d2) when compared with the DPP group. The difference in the renal injury was further reflected by the semiquantitative histological damage score (Figure 1C).

### QSYQ improves renal microvascular blood flow velocity, renal blood flow, and leukocyte adhesion

**Figure 2:**
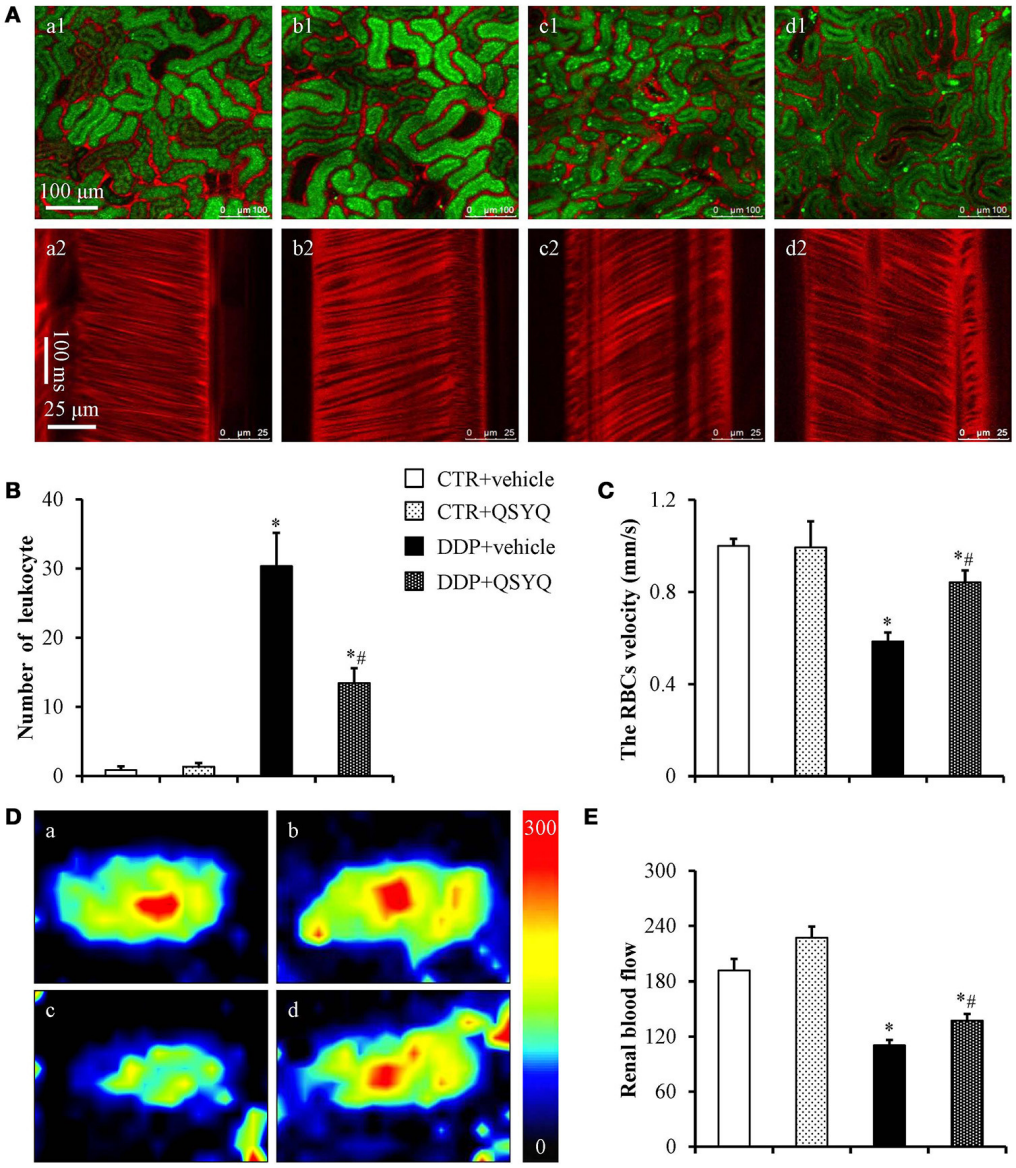
Effects of QSYQ on adherent leukocytes, the RBC flow velocity and renal blood flow in mice after DDP treatment. **(A)** *In vivo* imaging of renal adherent leukocytes **(a1–d1)** and RBC flow velocity **(a2–d2)** 72 h after DDP administration in CTR+vehicle (**a**, *N* = 6), CTR+QSYQ (**b**, *N* = 6), DDP+vehicle (**c**, *N* = 6), and DDP+QSYQ (**d**, *N* = 6) group. Red: Alexa fluor 647 labeled BSA showing blood vessels; Green: acridine orange showing renal tubule and adherent leukocytes. **(B)** Quantification of the adherent leukocytes. **(C)** Quantification of the microvascular blood flow. **(D)** Representative color images of renal blood flow acquired by laser Doppler perfusion imager in CTR+vehicle (**a**, *N* = 6), CTR+QSYQ (**b**, *N* = 6), DDP+vehicle (**c**, *N* = 6), and DDP+QSYQ (**d**, *N* = 6) group. **(E)** Quantification of the renal blood flow in different groups. ^*^P < 0.05 vs. CTR+vehicle group; ^#^*P* < 0.05 vs. DDP+vehicle group. Data are mean ± SE.

To address the effect of QSYQ pretreatment on DDP-induced renal microcirculation disturbance, we used *in vivo* imaging of renal cortex microvessels to measure microvascular blood flow velocity (Figure 2). The results showed that the blood flow velocity in the CTR+vehicle group and CTR+QSYQ group was similar to each other (Figures 2A, a2, b2,C). Compared to CTR+vehicle group, the microvascular blood flow velocity significantly decreased in DDP+vehicle group. However, QSYQ pretreatment protected the DDP-induced microvascular blood flow velocity decrease (Figures 2A, c2, d2,C). Besides, we used a laser Doppler perfusion imager to measure RBF (Figures 2D,E). Similarly, pretreatment with QSYQ significantly attenuated DDP-induced decrease in RBF. These results imply that QSYQ improves renal perfusion and DDP-induced renal microcirculatory disturbance. The leukocyte adhesion in renal cortex was also assessed with an intravital fluorescent microscope with the representative images in different groups displayed in Figure 2A. Pretreatment with QSYQ attenuated the number of adherent leukocytes. Figure 2B is the quantification of the adherent leukocytes, which confirmed the survey in Figure 2A.

### QSYQ improves mitochondrial respiratory chain complexes activities

**Figure 3:**
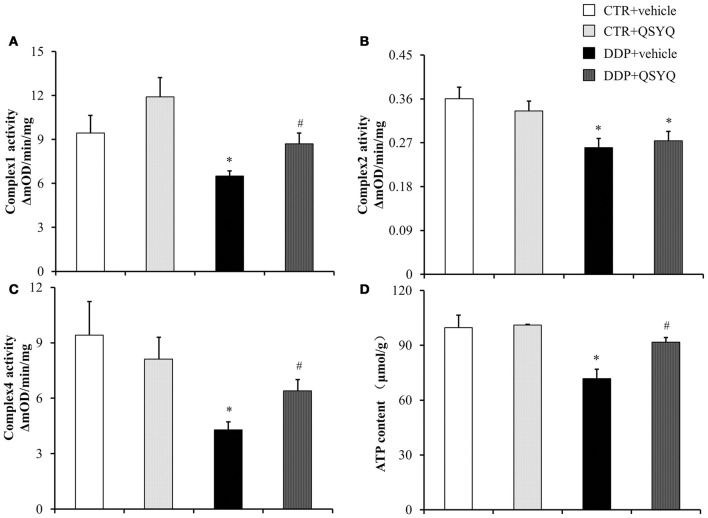
QSYQ protects DDP-induced mitochondria dysfunction. ELISA analysis was performed to determine **(A)** Complex I Activity, **(B)** Complex II activity, **(C)** Complex IV activity, and **(D)** ATP content in renal cortex from CTR+vehicle (*N* = 4), CTR+QSYQ (*N* = 4), DDP+vehicle (*N* = 6), and DDP + QSYQ (*N* = 6). ^*^P < 0.05 vs. CTR+vehicle group; ^#^*P* < 0.05 vs. DDP+vehicle group. Data are mean ± SE.

**Figure 4:**
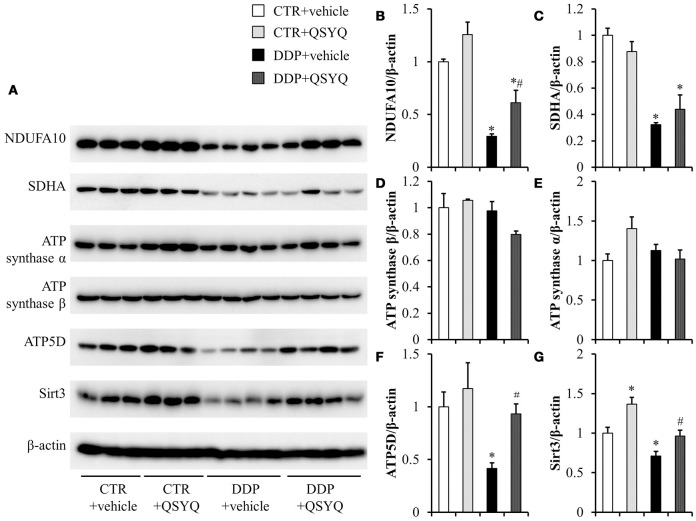
The effect of QSYQ on mitochondrial respiratory chain-related proteins in mice renal cortex after DDP treatment. **(A)** Representative western blot bands of NDUFA10, SDHA, ATP synthase α, ATP synthase β, ATP5D, and Sirt3 in different groups. **(B–G)** Quantitative analysis of the western blotting of NDUFA10, SDHA, ATP synthaseα, ATP synthase β, ATP5D, and Sirt3, respectively. CTR+vehicle: *N* = 3; CTR+QSYQ: *N* = 3; DDP+vehicle: *N* = 4;DDP + QSYQ: *N* = 4. ^*^P < 0.05 vs. CTR+vehicle group; ^#^*P* < 0.05 vs. DDP+vehicle group; Data are mean ± SE.

Mitochondrial dysfunction is known to be involved in DDP-induced acute kidney injury (Gordon and Gattone, 1986; Yang et al., 2014). We thus tested ATP content and the activities of mitochondrial respiratory chain complexes I, II, and IV by ELISA in renal cortex tissues of different groups (Figure 3). We found that ATP content decreased and activities of complexes I, II, and IV were suppressed to various degrees in the DDP+vehicle group compared with CTR+vehicle groups. QSYQ administration significantly restored ATP content and mitochondrial respiratory chain complex I and IV activities. Meanwhile, western blot showed that the expression of NADH:ubiquinone oxidoreductase subunit A10 (NDUFA10), succinate dehydrogenase complex flavoprotein subunit A (SDHA), ATP synthase δ-subunit (ATP5D), and Sirt3 decreased significantly after DDP treatment, while the expression of ATP synthase α and ATP synthase β had no significant change. QSYQ pretreatment restored the expression of NDUFA10, ATP5D, and Sirt3, but did not affect the expression of SDHA (Figure 4). These results indicated that pretreatment with QSYQ may attenuate DDP-induced nephrotoxicity via regulating mitochondrial respiratory chain.

### QSYQ ameliorates DDP-induced apoptosis and oxidative stress

**Figure 5:**
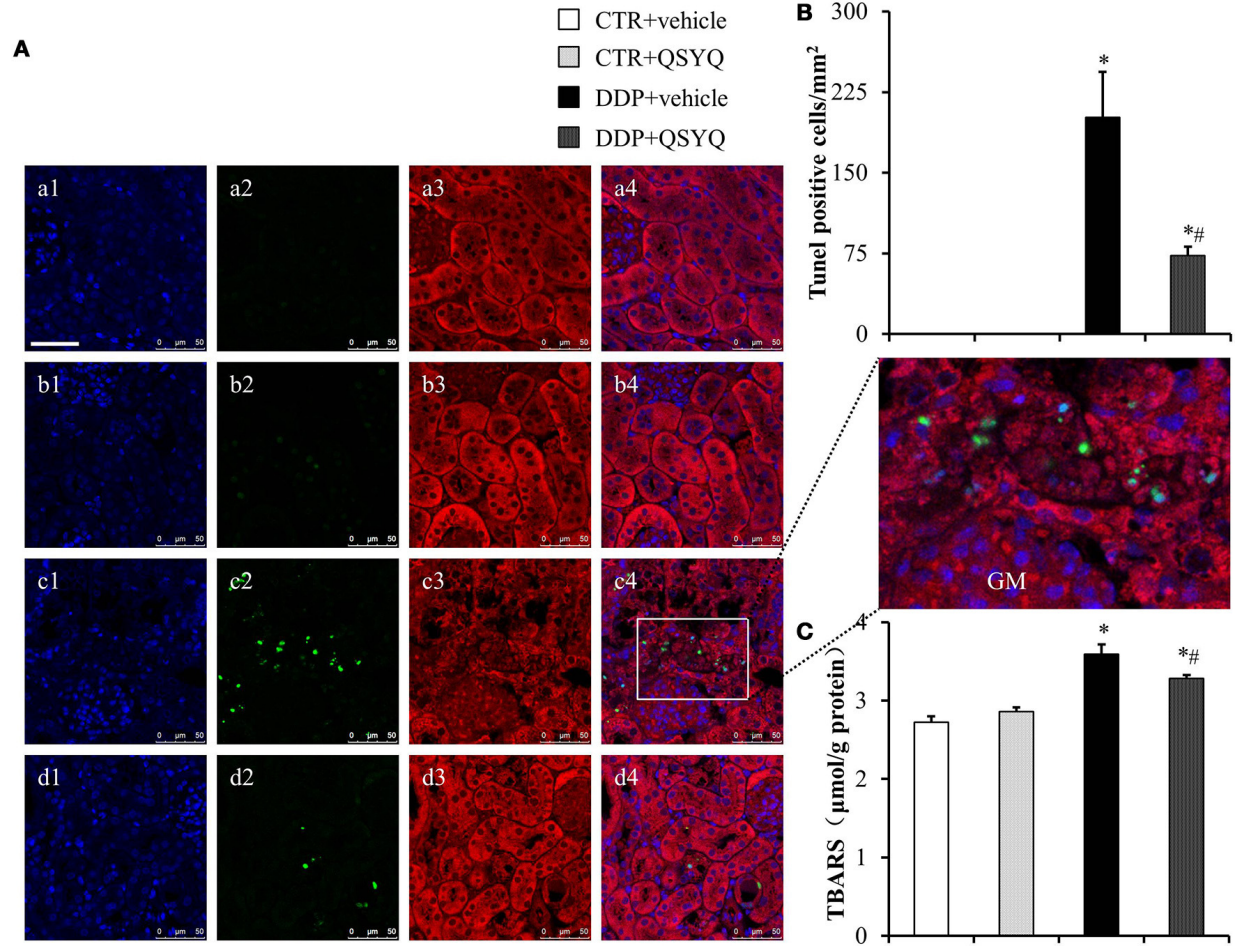
QSYQ protects DDP-induced oxidative stress and apoptosis in mice renal cortex. **(A)** Presented are the representative photographs of double staining of F-actin (3, red) and TUNEL(2, green) in CTR+vehicle **(a)**, CTR+QSYQ **(b)**, DDP+vehicle **(c)**, and DDP+QSYQ **(d)** group mice. Nucleus are stained with blue (1). GM: glomerulus. Bar = 50 μm. **(B)** Quantitative analysis of apoptosis cells among the various groups. CTR+vehicle: *N* = 3; CTR+QSYQ: *N* = 3; DDP+vehicle: *N* = 4;DDP + QSYQ: *N* = 4. ^*^P < 0.05 vs. CTR+vehicle group; #*P* < 0.05 vs. DDP+vehicle group; Data are mean ± SE. **(C)** Assessment of kidney thiobarbituric acid-reactive substances (TBARS). CTR+vehicle: *N* = 4; CTR+QSYQ: *N* = 4; DDP+vehicle: *N* = 6;DDP + QSYQ: *N* = 6. ^*^P < 0.05 vs. CTR+vehicle group; ^#^*P* < 0.05 vs. DDP+vehicle group; Data are mean ± SE.

**Figure 6:**
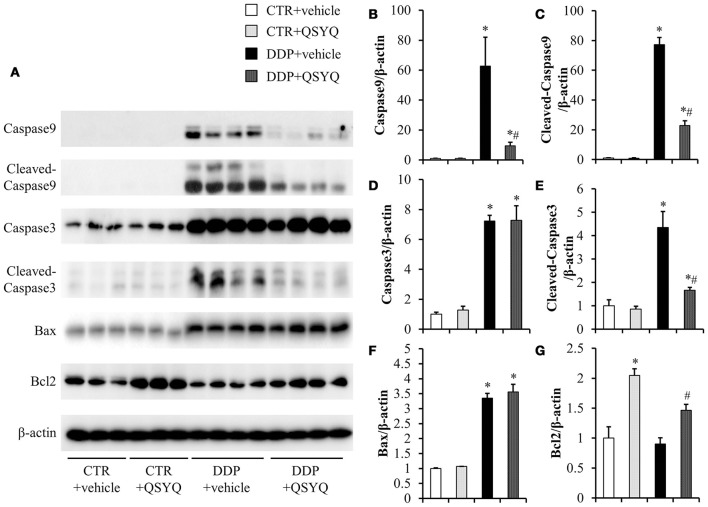
Assessment of apoptosis-related proteins in mice renal cortex from different groups. **(A)** Representative western blot bands of caspase9, cleaved-caspase9, caspase3, cleaved-caspase3, Bax, and Bcl2 in different groups. **(B–G)** Quantitative analysis of the western blotting of caspase9, cleaved-caspase9, caspase3, cleaved-caspase3, Bax, and Bcl2, respectively, in CTR+vehicle (*N* = 3), CTR+QSYQ (*N* = 3), DDP+vehicle (*N* = 4), and DDP + QSYQ (*N* = 4). ^*^P < 0.05 vs. CTR+vehicle group; ^#^*P* < 0.05 vs. DDP+vehicle group; Data are mean ± SE.

Accumulating evidence has demonstrated the pivotal role of mitochondria in DDP-induced nephropathy. Mitochondrial impairment not only mediates apoptosis, but also accounts for majority of ROS formation (Yang et al., 2014; Choi et al., 2015), both of which contribute to nephropathy. Therefore, we evaluated the effect of QSYQ pretreatment on DDP-induced oxidative stress, in view of the protection of QSYQ on DDP-caused mitochondria dysfunction, The level of kidney thiobarbituric acid-reactive substances (TBARS), an index of ROS generation, significantly increased after DDP treatment and this increase was significantly reduced by QSYQ pretreatment (Figure 5C). We next evaluated the effect of QSYQ pretreatment on DDP-induced apoptosis using TUNEL staining. As shown in Figures 5A,B, apoptotic cells were scarcely observed in the CTR+vehicle group and CTR+QSYQ group but increased significantly in the DDP+vehicle group. Noticeably, QSYQ pretreatment protected the apoptosis of renal tissue in the DDP+QSYQ group. In line with the results from TUNEL staining, the examination of expression of caspase9, cleaved-caspase9, cleaved-caspase3, Bcl2, and Bax by western blots (Figure 6) showed that DDP lead to an increase in proapoptotic caspase9, cleaved-caspase9, cleaved-caspase3, and Bax but a decrease in antiapoptotic Bcl2. With the exception of Bax, pretreatment with QSYQ inhibited all the abnormal expression of these apoptosis molecules. Interestingly, the expression of Bcl2 also increased in CTR+QSYQ group when compared to CTR+vehicle group. These results suggest that QSYQ could protect renal cells from DDP-induced apoptosis by a mechanism involving caspase9 and Bcl2.

## Discussion

In the present study, DDP exhibited apparent nephrotoxicity in mice, including the increase in plasma BUN and creatine, the leukocyte adhesion in renal cortex, the level of kidney oxidative stress, renal cell apoptosis, and a decrease in RBF and renal microvascular blood flow velocity. All the manifestations were significantly protected by QSYQ pretreatment. In addition, the results from the present study suggest that the disorder in mitochondrial respiratory chain may underlay the DDP nephrotoxicity, and this disorder is presumably prevented from by QSYQ pretreatment via the regulation of NDUFA10, ATP5D, and Sirt3.

The DDP nephrotoxicity has been noticed shortly after its application in clinic for treatment of cancer, which exhibits a spectrum of disorders with acute kidney injury (AKI) as the most serious presentation. In accordance with this commonly recognized manifestation, we observed in the present study a elevated level of urea and creatinine in plasma in mice following DDP administration, indicating the occurrence of AKI. Of the mechanisms so far proposed to underlay the DDP-induced AKI, inflammation is well accepted as an important player. The evidence supporting this notion mostly comes from the study on the response of inflammatory cytokines to DDP administration. The DDP increased necrosis factor (TNF)-α), interleukin (IL)-1β, IL-6, and transforming growth factor (TGF)-β1 have been reported in a number of publications (Miller et al., 2010). To attenuate the DDP-induced nephrotoxicity by inhibition of inflammation, a recently published study recommended to use thalidomide, which was found effective in animal model (Amirshahrokhi and Khalili, 2015) but still needs more study for clinic translation. In the present study, we observed a decreased renal blood flow, as well as a reduced red blood velocity and an increased leukocyte adhesion in kidney after DDP administration, which together suggest a disturbed microcirculation in kidney. As microcirculation is known to be sensitive to inflammation, this disturbed microcirculation most probably reflects the DDP-induced kidney inflammation. Interestingly, we found that QSYQ was effective in improvement of kidney blood flow and reduction of leukocytes adhesion, suggesting that the protective effect of QSYQ on the DDP-induced AKI is attributable to its role in normalizing microcirculation. This result is not unexpected considering the the herbs in QSYQ, which includes *S. miltiorrhiza* and *P. notoginseng* as major ingredients, both of which are demonstrated having potential to improve microcirculation (Han et al., 2008; Chen Y. Y. et al., 2015; Lv et al., 2017).

The role of mitochondria in DDP nephrotoxicity has attracted much attention. This is because DDP is hydrolyzed to generate a positively charged metabolite which preferentially accumulates within the negatively charged mitochondria. Moreover, mitochondrial DNA may be more susceptible than nuclear DNA to DDP-induced damage, due to less efficient DNA repair mechanisms (Miller et al., 2010; Yang et al., 2014). Another finding of the current study is the impairment of mitochondria in kidney tissue by DDP treatment. This result is consistent with some reports from others (Kruidering et al., 1997; Marullo et al., 2013; Galgamuwa et al., 2016). Importantly, we demonstrated that QSYQ may prevent all the manifestations of mitochondria impairment by DDP, along with elevating ATP level and attenuating oxidative stress and apoptosis, suggesting mitochondria as the target for QSYQ. However, the exact role of QSYQ-improved mitochondrial function in DDP nephrotoxicity needs to be explored by further studies. As a major source of energy, impairment of mitochondria may lead to ATP depletion, thus affects a diversity of cell function. To our knowledge, no study has been published to address the role of ATP depletion in DDP nephrotoxicity, although ATP is required for a normally functioning kidney. In contrast, the available data seem to point to an adverse effect of ATP in initiating DDP nephrotoxicity. The copper transporter CTR1 and organic cation transporter OCT2 are known to localize to the basolateral membrane of the proximal tubule epithelial cells, which uptake DDP into and damage the cells. Evidence shows that inhibition of the two transporters decreases the DDP nephrotoxicity. Normal function of the two transporters does not directly depend on ATP, but does depend on the plasma membrane potential created by an energy consumed process (Harrach and Ciarimboli, 2015). In this sense, the increase of ATP may not protect but rather promote the cells injury by DDP. Therefore, whether and how QSYQ-elevated ATP contributes to the observed attenuation of DDP nephrotoxicity remains an open question.

Another possibility is that QSYQ protected mitochondria from injury by DDP, diminishing the oxidative stress. Indeed, the leakage of electrons from the impaired mitochondrial respiratory chain represents a major intracellular source of free radicals (Santos et al., 2007). The contribution of oxidative stress to the DDP nephrotoxicity has been extensively investigated (Hajian et al., 2014), showing that mitochondrial targeted antioxidants successfully prevented oxidative stress and cell death in DDP-induced *in vivo* model of nephropathy (Mukhopadhyay et al., 2012). The present study revealed that the DDP-increased kidney ROS generation was significantly protected by QSYQ pretreatment, suggesting a likely involvement of antioxidation in the effect of QSYQ on DDP nephrotoxicity.

Besides, the intrinsic pathway of apoptosis is centered on mitochondria and plays a critical role in DDP nephrotoxicity (Park et al., 2002; Jiang et al., 2009), in which caspase9 is a key protein and promoter. Activation of the caspase9 is dependent primarily on mitochondrial signaling pathways regulated by the members of the Bcl2 family such as Bax, activation of which leads to alterations in mitochondrial permeability, release of cytochrome c, and activation of caspase9, resulting in activation of downstream of caspases, including caspase 3/6/7, and eventually cell apoptosis (Li et al., 2017). The present study indicated that QSYQ may attenuate kidney injury via inhibiting the mitochondrial pathway of apoptosis.

Taken together, the present study showed mitochondria as a central player in QSYQ attenuating DDP nephrotoxicity by inhibiting oxidative stress and apoptosis elicited by DDP. Nevertheless, the detailed mechanism for the effect of QSYQ needs to be clarified by more studies. In addition, the paradox encountered in this field is that the medicine able to reduce adverse side effect of an anticancer drug frequently also lessens the effect of this drug for treatment of cancer. Therefore, the clinic feasibility of QSYQ for cancer patients undergoing DDP treatment requires validation.

In conclusion, the present study evaluated the effects of QSYQ on DDP-induced nephrotoxicity, showing the ability of QSYQ to attenuate the DDP-induced renal dysfunction and microcirculatory disturbance. The protective effect of QSYQ is correlated with its potential to modulate mitochondrial respiratory chain, upregulating NDUFA10, SDHA, and ATP5D leading to a decrease in oxidative stress and apoptosis. The results provide a novel option for dealing with DDP-induced nephrotoxicity. Further studies are needed to verify the feasibility of QSYQ as an effective nephroprotective agent in chemotherapy regimens that include DDP.

## Author contributions

LZ performed the research, analyzed the data and wrote the manuscript. KS, Y-YG and Y-YL contributed to animal experiments. X-HW and LY contributed to immunochemistry analysis and cell culture. C-SP contributes to western blotting. C-SW contributes to other experiments. J-YF and J-YH revised the manuscript. J-YH designed and funded the research, interpreted the data, and finally approved the submission of this manuscript. All authors have read and agreed with the manuscript.

### Conflict of interest statement

The authors declare that the research was conducted in the absence of any commercial or financial relationships that could be construed as a potential conflict of interest.

## Abbreviations

AKI: acute kidney injury
ANOVA: analysis of variance
ATP: adenosine triphosphate
ATP5D: ATP synthase δ-subunit
Bax: B-cell lymphoma-2 associated X protein
Bcl-2: B-cell lymphoma-2
BSA: bovine serum albumin
BUN: blood urea nitrogen
CTR: control
DDP: cis-diamminedichloroplatinum
DNA: deoxyribonucleic acid
ELISA: enzyme linked immunosorbent assay
NDUFA10: NADH:ubiquinone oxidoreductase subunit A10
PAS: periodic acid schiff
PFA: paraformaldehyde
QSYQ: QiShenYiQi Pills
RBC: red blood cell
RBF: renal blood flow
ROS: reactive oxygen species
SDHA: succinate dehydrogenase complex flavoprotein subunit A
TBARS: thiobarbituric acid reactive substances
TUNEL: terminal deoxynucleotidyl transferase-mediated dUTP nick end labeling.

## References

[B1] Amirshahrokhi K. Khalili A. R. (2015). Thalidomide ameliorates cisplatin-induced nephrotoxicity by inhibiting renal inflammation in an experimental model. Inflammation 38, 476–484. 10.1007/s10753-014-9953-7 24950782

[B2] Arany I. Safirstein R. L. (2003). Cisplatin nephrotoxicity. Semin. Nephrol. 23, 460–464. 10.1016/S0270-9295(03)00089-5 13680535

[B3] Cahoon J. M. Olson P. R. Nielson S. Miya T. R. Bankhead P. McGeown J. G.. (2014). Acridine orange leukocyte fluorography in mice. Exp. Eye Res. 120, 15–19. 10.1016/j.exer.2013.12.002 24333760 PMC3943592

[B4] Chaigneau E. Oheim M. Audinat E. Charpak S. (2003). Two-photon imaging of capillary blood flow in olfactory bulb glomeruli. Proc. Natl. Acad. Sci. U.S.A. 100, 13081–13086. 10.1073/pnas.2133652100 14569029 PMC240748

[B5] Chen J. R. Wei J. Wang L. Y. Zhu Y. Li L. Olunga M. A.. (2015). Cardioprotection against ischemia/reperfusion injury by QiShenYiQi Pill(R) via ameliorate of multiple mitochondrial dysfunctions. Drug Des. Dev. Ther. 9, 3051–3066. 10.2147/DDDT.S82146 26109848 PMC4474392

[B6] Chen Y. Y. Li Q. Pan C. S. Yan L. Fan J. Y. He K.. (2015). QiShenYiQi Pills, a compound in Chinese medicine, protects against pressure overload-induced cardiac hypertrophy through a multi-component and multi-target mode. Sci. Rep. 5:11802. 10.1038/srep11802 26136154 PMC4488877

[B7] Choi Y. M. Kim H. K. Shim W. Anwar M. A. Kwon J. W. Kwon H. K.. (2015). Mechanism of cisplatin-induced cytotoxicity is correlated to impaired metabolism due to mitochondrial ROS generation. PLoS ONE 10:e0135083. 10.1371/journal.pone.0135083 26247588 PMC4527592

[B8] Dilruba S. Kalayda G. V. (2016). Platinum-based drugs: past, present and future. Cancer Chemother. Pharmacol. 77, 1103–1124. 10.1007/s00280-016-2976-z 26886018

[B9] Dunn K. W. Sandoval R. M. Kelly K. J. Dagher P. C. Tanner G. A. Atkinson S. J.. (2002). Functional studies of the kidney of living animals using multicolor two-photon microscopy. Am. J. Physiol. Cell Physiol. 283, C905–C916. 10.1152/ajpcell.00159.2002 12176747

[B10] Galgamuwa R. Hardy K. Dahlstrom J. E. Blackburn A. C. Wium E. Rooke M.. (2016). Dichloroacetate prevents cisplatin-induced nephrotoxicity without compromising cisplatin anticancer properties. J. Am. Soc. Nephrol. 27, 3331–3344. 10.1681/ASN.2015070827 26961349 PMC5084882

[B11] Gordon J. A. Gattone V. H. II. (1986). Mitochondrial alterations in cisplatin-induced acute renal failure. Am. J. Physiol. 250(6 Pt 2), F991–F998. 3717354 10.1152/ajprenal.1986.250.6.F991

[B12] Hajian S. Rafieian-Kopaei M. Nasri H. (2014). Renoprotective effects of antioxidants against cisplatin nephrotoxicity. J. Nephropharmacol. 3, 39–42. 28197460 PMC5297526

[B13] Han J. Y. Fan J. Y. Horie Y. Miura S. Cui D. H. Ishii H.. (2008). Ameliorating effects of compounds derived from *Salvia miltiorrhiza* root extract on microcirculatory disturbance and target organ injury by ischemia and reperfusion. Pharmacol. Ther. 117, 280–295. 10.1016/j.pharmthera.2007.09.008 18048101

[B14] Han J. Y. Li Q. Ma Z. Z. Fan J. Y. (2017). Effects and mechanisms of compound Chinese medicine and major ingredients on microcirculatory dysfunction and organ injury induced by ischemia/reperfusion. Pharmacol. Ther. 177, 146–173. 10.1016/j.pharmthera.2017.03.005 28322971

[B15] Harrach S. Ciarimboli G. (2015). Role of transporters in the distribution of platinum-based drugs. Front. Pharmacol. 6:85. 10.3389/fphar.2015.00085 25964760 PMC4408848

[B16] Jia Z. Wang N. Aoyagi T. Wang H. Liu H. Yang T. (2011). Amelioration of cisplatin nephrotoxicity by genetic or pharmacologic blockade of prostaglandin synthesis. Kidney Int. 79, 77–88. 10.1038/ki.2010.331 20844471

[B17] Jiang M. Wang C. Y. Huang S. Yang T. Dong Z. (2009). Cisplatin-induced apoptosis in p53-deficient renal cells via the intrinsic mitochondrial pathway. Am. J. Physiol. Renal Physiol. 296, F983–F993. 10.1152/ajprenal.90579.2008 19279129 PMC2681364

[B18] Kruidering M. Van de Water B. de Heer E. Mulder G. J. Nagelkerke J. F. (1997). Cisplatin-induced nephrotoxicity in porcine proximal tubular cells: mitochondrial dysfunction by inhibition of complexes I to IV of the respiratory chain. J. Pharmacol. Exp. Ther. 280, 638–649. 9023274

[B19] Li P. Zhou L. Zhao T. Liu X. Zhang P. Liu Y.. (2017). Caspase-9: structure, mechanisms and clinical application. Oncotarget 8, 23996–24008. 10.18632/oncotarget.15098 28177918 PMC5410359

[B20] Li Y. C. Liu Y. Y. Hu B. H. Chang X. Fan J. Y. Sun K.. (2012). Attenuating effect of post-treatment with QiShen YiQi Pills on myocardial fibrosis in rat cardiac hypertrophy. Clin. Hemorheol. Microcirc. 51, 177–191. 10.3233/CH-2011-1523 22240383

[B21] Lin S. Q. Wei X. H. Huang P. Liu Y. Y. Zhao N. Li Q.. (2013). QiShenYiQi Pills(R) prevent cardiac ischemia-reperfusion injury via energy modulation. Int. J. Cardiol. 168, 967–974. 10.1016/j.ijcard.2012.10.042 23168012

[B22] Lv S. C. Wu M. Li M. Wang Q. Wang X. J. Zhang A.. (2017). Effect of QiShenYiQi pill on myocardial collagen metabolism in experimental autoimmune myocarditis rats. Biomed. Pharmacother. 88, 894–901. 10.1016/j.biopha.2017.01.096 28178619

[B23] Marullo R. Werner E. Degtyareva N. Moore B. Altavilla G. Ramalingam S. S.. (2013). Cisplatin induces a mitochondrial-ROS response that contributes to cytotoxicity depending on mitochondrial redox status and bioenergetic functions. PLoS ONE 8:e81162. 10.1371/journal.pone.0081162 24260552 PMC3834214

[B24] Miller R. P. Tadagavadi R. K. Ramesh G. Reeves W. B. (2010). Mechanisms of Cisplatin nephrotoxicity. Toxins 2, 2490–2518. 10.3390/toxins2112490 22069563 PMC3153174

[B25] Mukhopadhyay P. Horváth B. Zsengellér Z. Zielonka J. Tanchian G. Holovac E.. (2012). Mitochondrial-targeted antioxidants represent a promising approach for prevention of cisplatin-induced nephropathy. Free Radic. Biol. Med. 52, 497–506. 10.1016/j.freeradbiomed.2011.11.001 22120494 PMC3253235

[B26] Nowak G. (2002). Protein kinase C-α and ERK1/2 mediate mitochondrial dysfunction, decreases in active Na^+^ transport, and cisplatin-induced apoptosis in renal cells. J. Biol. Chem. 277, 43377–43388. 10.1074/jbc.M206373200 12218054 PMC1948818

[B27] Park M. S. De Leon M. Devarajan P. (2002). Cisplatin induces apoptosis in LLC-PK1 cells via activation of mitochondrial pathways. J. Am. Soc. Nephrol. 13, 858–865. 11912244 10.1681/ASN.V134858

[B28] Sánchez-González P. D. López-Hernández F. J. López-Novoa J. M. Morales A. I. (2011). An integrative view of the pathophysiological events leading to cisplatin nephrotoxicity. Crit. Rev. Toxicol. 41, 803–821. 10.3109/10408444.2011.602662 21838551

[B29] Santos N. A. Catão C. S. Martins N. M. Curti C. Bianchi M. L. Santos A. C. (2007). Cisplatin-induced nephrotoxicity is associated with oxidative stress, redox state unbalance, impairment of energetic metabolism and apoptosis in rat kidney mitochondria. Arch. Toxicol. 81, 495–504. 10.1007/s00204-006-0173-2 17216432

[B30] Tang D. X. Zhao H. P. Pan C. S. Liu Y. Y. Wei X. H. Yang X. Y.. (2013). QiShenYiQi Pills, a compound chinese medicine, ameliorates doxorubicin-induced myocardial structure damage and cardiac dysfunction in rats. Evid. Based Complement. Alternat. Med. 2013:480597. 10.1155/2013/480597 23533487 PMC3600323

[B31] Tang P. Zhang Y. Chen C. Ji X. Ju F. Liu X.. (2015). *In vivo* two-photon imaging of axonal dieback, blood flow, and calcium influx with methylprednisolone therapy after spinal cord injury. Sci. Rep. 5:9691. 10.1038/srep09691 25989524 PMC4437044

[B32] Wang H. Jia Z. Sun J. Xu L. Zhao B. Yu K.. (2015). Nitrooleic acid protects against cisplatin nephropathy: role of COX-2/mPGES-1/PGE2 cascade. Mediators Inflamm. 2015:293474. 10.1155/2015/293474 25861160 PMC4377489

[B33] Wang Y. Zhao X. Gao X. Nie X. Yang Y. Fan X. (2011). Development of fluorescence imaging-based assay for screening cardioprotective compounds from medicinal plants. Anal. Chim. Acta 702, 87–94. 10.1016/j.aca.2011.06.020 21819864

[B34] Yang Y. Liu H. Liu F. Dong Z. (2014). Mitochondrial dysregulation and protection in cisplatin nephrotoxicity. Arch. Toxicol. 88, 1249–1256. 10.1007/s00204-014-1239-1 24859930 PMC4274771

